# Vascular dysfunction and dyslipidemia in multiple sclerosis: are they correlated with disease duration and disability status?

**DOI:** 10.1186/s43044-022-00244-2

**Published:** 2022-02-11

**Authors:** Hesham Boshra, Marina Awad, Mona Hussein, Ehab Elyamani

**Affiliations:** 1 Department of Cardiology, Beni-Suef University, Beni-suef, Egypt; 2 Department of Neurology, Beni-Suef University, Beni-suef, 62511 Egypt

**Keywords:** Multiple sclerosis, EDSS, Arterial stiffness, Pulse wave velocity, Lipid profile

## Abstract

**Background:**

There is strong evidence that vascular dysfunction is considered one of the possible causes of morbidity and mortality in patients suffering from multiple sclerosis (MS). This work aimed at assessing the arterial function and serum lipids in MS patients and correlating them with clinical and radiological findings.

**Results:**

This case–control study included 50 patients with MS and 50 age- and sex-matched controls. The arterial function was significantly reduced in MS patients, confirmed by significantly higher pulse wave velocity (PWV) and augmentation index (AIX), while the carotid IMT did not show significant difference between the two groups with no plaques in any of our patients. A significant positive correlation was found between PWV and both disease duration and disability. MS patients had significantly higher serum levels of T-cholesterol and triglycerides, and significantly lower serum levels of HDL-cholesterol, compared to controls. No significant correlation was found between serum lipids and either disease duration or disability.

**Conclusions:**

There was a significant impairment in arterial function (assessed by the brachial cuff-based method via Mobil-O-Graph device) in MS patients compared to controls. Such impairment was significantly correlated with both disease duration and disability. MS patients had also significantly higher levels of T-cholesterol and triglycerides, compared to controls with no significant correlation between serum lipids and either disease duration or disability.

## Background

Multiple sclerosis (MS) is a chronic immune-mediated neurological disorder with recurrent episodes of inflammation, demyelination of the central nervous system and subsequent axonal degeneration [1]. It is considered the main reason of neurological disability in young populations that affects women 2 times more often than men [2]. The clinical course of MS varies; more than 80% of patients develop attacks of disability followed by periods of either partial or complete recovery (relapsing–remitting), while about15% of patients have a more progressive course without remissions [3].

MS patients were reported to have a higher mortality rate in comparison with the general population, and this was attributed to many factors including the higher incidence of cardiovascular diseases among this population [4]. There is strong evidence that MS was associated with an increased risk for stroke, myocardial infarction and heart failure early after MS diagnosis [5].

The main mechanisms of vascular dysfunction in MS have not been well understood yet; however, the status of chronic inflammation and autoimmunity, oxidative stress and the cardiovascular autonomic dysregulation in MS patients were suggested to be possible mechanisms. Inflammation has a major role in atherosclerosis, arteriosclerosis and endothelial dysfunction. It also enables plaque rupture increasing the risk of acute coronary syndromes [6]. Oxidative stress was also reported to increase several times in MS patients, leading to endothelial dysfunction, arterial remodeling and stiffness, and overt atherosclerosis [7]. Additionally, the cardiovascular autonomic dysregulation, frequently occurring in MS patients, might contribute to increasing the risk of microvascular disease and coronary vasospasm [8]. Other factors that might play a role in vascular dysfunction in MS patients are related to the increased incidence of many cardiovascular risk factors in the MS patients including diabetes, obesity, smoking, physical inactivity, hyperhomocysteinemia, insulin resistance, low levels of vitamin D, psychological stress and the procoagulant status of MS [9].

Despite the importance of research in this field, assessment of vascular function was not extensively performed in those patients and little data are available about the differential degrees of atherosclerosis between MS patients and the general population. This work aimed at assessment of the arterial function and serum lipids in MS patients and to correlate them with disease duration and severity. This might aid in the early detection of vascular dysfunction and dyslipidemia in MS patients, and consequently improving their vascular health.

## Methods

*Study design and study population*: A case–control study that included 50 patients diagnosed with relapsing–remitting multiple sclerosis and 50 age- and sex-matched controls. MS patients were recruited in the period from January to October 2020. The healthy control subjects were selected to have an age range and sex distribution close to the included patients. A written informed consent was taken from all patients and controls, or their relatives. The study was conducted in accordance with the Declaration of Helsinki. The ethical committee approved this study.

*Inclusion criteria*: Patients diagnosed as having relapsing–remitting multiple sclerosis (RRMS) according to the International Panel on Diagnosis of Multiple Sclerosis “McDonald’s criteria 2017” [10]. Patients were assessed in the remission state (at least one month after the last relapse). The age range for the patients, included in the study, was between 15 and 45 years.

*Exclusion criteria*: The following patients were excluded from the study: hypertensive or diabetic patients, patients with known cardiovascular disease, patients with associated autoimmune disease, and patients with known pulmonary, hepatic, renal, hematological or endocrinal disease. Pregnant patients were also excluded from the study.

The included participants were subjected to the following.

1. *History taking from MS patients regarding*: The total number of relapses, disease duration and the prescribed disease-modifying drugs (DMDs).
2. *Clinical assessment of all participants including*: Blood pressure, heart rate, height, weight, body mass index (BMI) and body surface area.
3. *Assessment of neurological disability for MS patients using Expanded Disability Status Scale (EDSS)*: The evaluated functional systems were pyramidal, cerebellar, cerebral, sensory and visual. The score of this scale ranges from zero (normal) to 10 (death) [11].
4. *Radiological assessment using magnetic resonance imaging (MRI) on the brain and spinal cord*: To detect size and site of MS plaques in addition to lesion load and to exclude other structural brain or spinal cord lesions.
5. *Measurement of peripheral pulse wave velocity and augmentation index*: Brachial cuff-based method was used in this study via (Mobil-O-Graph, I.E.M. Stolberg, Germany) with its analysis software Hypertension Management Software Client–Server (HMS-CS 4.3). It is a noninvasive tool for the assessment of a range of central arterial indices, approved by the European Society of Cardiology (ESC) and the Food and Drug Administration (FDA) [12]. All of the included patients and controls were asked to rest in a quiet room for about 5 min before the measurement. Smoking, uptake of vasoactive medications, caffeine, and alcohol were not allowed 4–6 h before the examination. All participants were examined in the sitting position with suitable cuffs wrapped around their non-dominant arm. The device performs a brachial oscillometric BP measurement, and then, it records the brachial pulse waveforms. The aortic pulse waveform is generated from the brachial pressure waveform using the ARCSolver algorithm of the device. The software of the Mobil-O-Graph device performs wave-separation analysis by decomposing the aortic pulse waveform into forward- and backward-traveling pulse waves with the use of a physiologic aortic flow waveform. The Mobil-O-Graph derived PWV is an indirect estimate of large-artery stiffness based on mathematical models incorporating several parameters derived from pulse wave analysis and wave-separation analysis. The augmentation index (AIX) was calculated as the ratio of the amplitude of the pressure wave above its systolic shoulder (i.e., the difference between the early and late systolic peaks of the arterial waveform) to the total pulse pressure and was expressed as a percentage. Data are transferred directly via Bluetooth technology after completion of the measurement. The ARCSolver algorithm provides equivalent performance to that of the SphygmoCor device and also to Central BP and its estimates, measured invasively [13, 14].
6. *Measurement of carotid intima-media thickness*: All measurements were done in a quiet room with a stable temperature with the patient in a supine position with a hyperextended neck, after at least 10 min of rest. The study was done using the ultrasound machine (Vivid S5), with an 8 MHz linear array probe. A high-resolution B-mode system was used. The common carotid artery (CCA) was identified in the transverse plane and scanned from its origin till its bifurcation, from multiple angles, to optimize the detection of non-obstructive plaques. Focus depth, frame rate and gain settings were adjusted optimally, and then, sequences of images and videos were obtained from CCA, the site of CCA bifurcation, and ICA bulb longitudinal views, and stored. During offline analysis, far wall carotid IMT was measured, at end of diastole, in the centimeter proximal to the carotid bifurcation, and at the site of carotid bulb. Three measurements were obtained from the CCA IMT on each side, and the mean IMT of these values was calculated.
7. *Screening for carotid plaques*: To improve the role of carotid IMT for the prediction of CV risk, we added screening of CCA and carotid bulb for plaques. A scan ranging from anterior to posterior angles and imaging the near and far walls of the CCA, CCA bifurcation site, and ICA bulb were done. The 2020 American consensus of use of carotid ultrasonography suggested that the thickness of a carotid arterial plaque lesion was chosen as the initial measure to define plaque. They also suggested a CIMT of more than 1.5 mm may be considered as a significant plaque lesion for patients below 65 years of age [15].
8. *Laboratory work*: Fasting peripheral blood samples were collected from all patients and controls. The samples were immediately centrifuged at 3000 g for 15 min and measured by the spectrophotometer device for cholesterol, triglycerides and HDL-C. LDL-C was measured indirectly by the Friedewald formula [16]:
  $${\text{LDL - C}}\;{\text{(mg/dl)}} = {\text{Total cholesterol}}{-}{\text{HDL - C}}{-}({\text{triglycerides/5}}).$$

### Intra- and inter-observer variability

Intra-observer and inter-observer variability for the carotid ultrasound and arterial stiffness parameters were examined in 10 randomly chosen patients. For intra-observer variability, the same reviewer re-measured the parameters after the initial measurement. Inter-observer variability was measured by a second reviewer who was blinded to the clinical history.

### Statistical analysis

Statistical analysis was done with SPSS software version 20. Quantitative variables were expressed as mean and standard deviation (SD). Categorical variables were expressed as number and percentages. Independent samples *T* test was used to compare between MS patients and controls in the quantitative variables, while Chi-square test was used to compare to compare between MS patients and controls in categorical variables. Pearson’s correlation was used to describe the association between the quantitative variables. *P*-values ≤ 0.05 (two-sided) were considered statistically significant.

## Results

This study is a case–control study conducted on fifty MS patients and fifty healthy controls. The baseline characteristics of patients and controls are demonstrated in Table 1. The MS patients and controls did not differ in age, sex, BMI, BSA, SBP, DBP, HR, or smoking status. The neurological and radiological characteristics of MS patients, in addition to the current DMDs, are demonstrated in Table 2.

**Table 1:** General characteristics of MS patients and controls

	Patients (*n* = 50)	Controls (*n* = 50)	*P*-value
Age in years [mean (SD)]	32 (8.47)	29 (7.51)	0.107
*Sex*			
Males [*n* (%)]	18 (36%)	16 (32%)	0.6
Females [*n* (%)]	32 (64%)	34 (68%)	
BMI (kg/m^2^) [mean (SD)]	26.5 (6.16)	26 (4.17)	0.723
BSA (m^2^) [mean (SD)]	1.7 (0.1)	1.7 (0.2)	0.71
SBP (mmhg) [mean (SD)]	118(8.97)	116 (6.66)	0.227
DBP (mmhg) [mean (SD)]	76 (7.5)	74.22 (6.5)	0.202
HR (beats/min) [mean (SD)]	84 (13.26)	82.7 (9.37)	0.55
*Smoking*			
Smokers [*n* (%)]	8 (16%)	8 (16%)	1
Non-smokers [*n* (%)]	42 (84%)	42 (84%)	

BMI, body mass index; BSA, body surface area; DBP, diastolic blood pressure; HR, heart rate; and SBP, systolic blood pressure

*P*-value ˃0.05 is considered statistically insignificant

**Table 2:** Neurological and radiological characteristics of MS patients

	Patients (*n* = 50)
Disease duration in years [mean (SD)]	4.29 (3.61)
EDSS [mean (SD)]	3.25 (1.47)
Total number of relapses [mean (SD)]	3.38 (3.61)
MRI lesion load [mean (SD)]	9.72 (6.66)
*DMDs*	
No DMDs [*n* (%)]	28 (56%)
Interferon beta [*n* (%)]	18 (36%)
Fingolimod [*n* (%)]	1 (2%)
Cyclophosphamide [*n* (%)]	1 (2%)
Azathioprine [*n* (%)]	1 (2%)
Rituximab [*n* (%)]	1 (2%)

DMDs, disease-modifying drugs; EDSS, Expanded Disability Status Scale; and MRI, magnetic resonance imaging

The MS patients had significantly reduced vascular function, compared to controls, as shown by a significantly higher peripheral PWV (5.6 vs 5.2, *P*-value = 0.001) and augmentation index (26.5 vs 19, *P*-value < 0.001). There was no statistically significant difference between the two groups as regards the right or left CCA IMT. None of our patients had a carotid plaque (Table 3).

**Table 3:** Vascular function of patients with multiple sclerosis and control subjects

	Patients (*n* = 50) mean (SD)	Controls (*n* = 50) mean (SD)	*P*-value
Peripheral PWV (m/s)	5.61 (0.58)	5.23 (0.505)	0.001*
Augmentation Index (%)	26.5 (10)	19.28 (7.29)	< 0.001*
Mean RT CCA IMT (mm)	0.45 (0.08)	0.42 (0.09)	0.122
Mean LT CCA IMT (mm)	0.45 (0.098)	0.47 (0.104)	0.315
RT carotid bulb IMT (mm)	0.81 (0.18)	0.67 (0.096)	< 0.001*
LT carotid bulb IMT (mm)	0.84 (0.2)	0.72 (0.1)	0.001*

IMT, intima-media thickness; LT CCA, left common carotid artery; PWV, pulse wave velocity; and RT CCA, right common carotid artery

*P-value < 0.05 is considered statistically significant

**Fig. 1:**
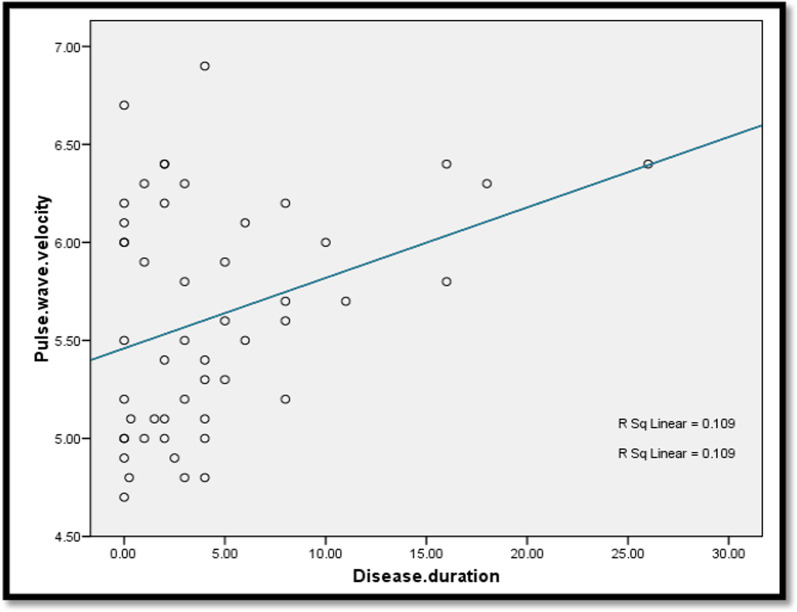
Correlation between disease duration (years) and peripheral PWV (m/s). PWV, pulse wave velocity

**Fig. 2:**
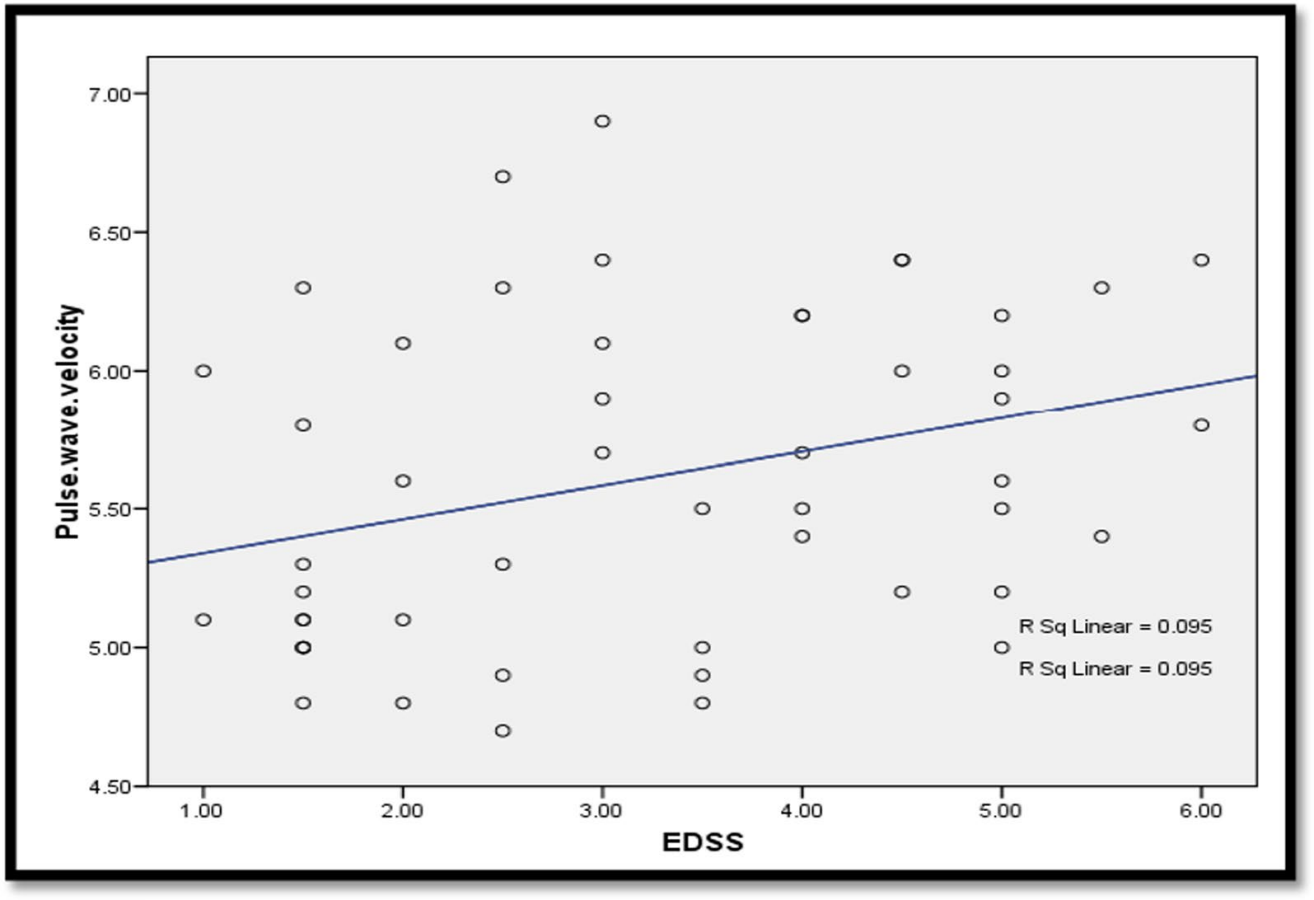
Correlation between EDSS and peripheral PWV (m/s). EDSS, Expanded Disability Status Scale; PWV, pulse wave velocity

There was a statistically significant positive correlation between age and PWV (*r* = 0.875, *P*-value ≤ 0.001). A statistically significant positive correlation was also found between PWV and both disease duration and EDSS score (*P*-value = 0.019, 0.029, respectively) (Figs. 1, 2). Also, there was a statistically significant positive correlation between the left CCA IMT and EDSS score (*P*-value = 0.029), while there was no statistically significant correlation between PWV and the total number of relapses or the MRI lesion load (*P*-value = 0.066, 0.982, respectively).

Regarding the lipid profile of MS patients and controls, MS patients had significantly higher serum levels of T-cholesterol (165.8 vs 151.5, *P*-value < 0.001), triglycerides (140 vs 120, *P*-value = 0.025), compared to controls. They also had insignificantly higher serum levels of low-density and very-low-density lipoprotein cholesterol, compared to controls. On the other hand, serum level of HDL-cholesterol was significantly lower in MS patients, compared to controls (46 vs 49.7, *P*-value = 0.007) (Table 4). There was no statistically significant correlation between T-cholesterol, triglycerides, or LDL-cholesterol in MS patients and either disease duration or disability.

**Table 4:** Serum lipids of patients with multiple sclerosis and control subjects

	Patients (*n* = 50) mean (SD)	Controls (*n* = 50) mean (SD)	*P*-value
T-cholesterol (mg/dl)	165.86 (41.13)	151.46 (26.66)	0.041*
TGs (mg/dl)	139.95 (57.54)	120.18 (20.13)	0.025*
HDL-C (mg/dl)	46.95 (6.3)	49.77 (3.49)	0.007*
LDL-C (mg/dl)	90.23 (39.53)	78.54 (27.39)	0.089
VLDL-C (mg/dl)	28.35 (11.55)	26.58 (7)	0.357

HDL-C, high-density lipoprotein cholesterol; LDL-C, low-density lipoprotein cholesterol; T-cholesterol, total cholesterol; TG, triglycerides; and VLDL, very-low-density lipoproteins

*P-value < 0.05 is considered statistically significant

To study the effect of DMDs on vascular function and lipid profile in MS patients, we compared MS patients who were on DMDs (*n* = 22) to those who did not receive DMDs (*n* = 28) regarding the parameters of vascular function and lipid profile, but there was no statistically significant difference between the two groups.

## Discussion

Recent population studies showed that MS was associated with an almost threefold increase in the risk of death [17] and this may be attributed to multiple factors including cardiovascular co-morbidities [18]. There is conflicting data about the vascular dysfunction in MS patients and its correlation with disease duration or severity. So, our work aimed at assessing vascular function and serum lipids in MS patients and correlating them with the neurological disability and imaging findings.

We found significantly impaired vascular function in patients with MS, compared to controls, shown by significantly higher peripheral PWV (*P*-value = 0.001) and augmentation index (*P*-value < 0.001) in MS patients, compared to controls, but we did not find a statistically significant difference in CCA IMT between the two groups. None of our patients had a carotid plaque. We also found a statistically significant positive correlation between PWV and both disease duration and EDSS score (*P*-value = 0.019, 0.029, respectively).

Similar to our findings, Talaat et al. assessed brachial–ankle PWV and carotid IMT in MS patients. They found that brachial–ankle PWV was significantly higher in MS patients compared to controls (*P*-value = 0.014). On the other hand, they found no statistically significant difference between patients and controls as regards carotid IMT. The investigators reported no statistically significant correlations between brachial–ankle PWV or carotid IMT, and EDSS scores in MS patients, compared to our current study which found a significant correlation between PWV and EDSS score. The authors explained the reported reduced arterial compliance in their MS patients by the status of chronic inflammation that may be a contributing factor to atherosclerosis initiation and progression [19].

Moreover, Ranadive et al. assessed central PWV and augmentation index using SphygmoCor device, in addition to carotid IMT, in MS patients. Physical activity was also measured by the ActiGraph single-axis accelerometer. The authors reported significantly higher central PWV in MS patients, compared to controls (*P*-value < 0.05), but there was no significant difference in carotid IMT between the two groups. In addition, physical activity was negatively correlated with central PWV. They concluded that physical inactivity may have accounted for some of the arterial dysfunction reported in their MS patients [20].

The vascular dysfunction in MS patients may be explained by the impact of chronic inflammation on the cardiovascular system. Strong evidence indicates that inflammation plays a major role in the formation and destabilization of atherosclerotic plaques, leading to acute cardiovascular events [6]. The dysregulated pro-inflammatory cytokines in MS, including IL-1, IL-6, TNF-ƒÑ, or IFN-ƒ×, were thought to be involved in inducing atherosclerosis in those patients [21]. Moreover, the reported increase in oxidative stress in MS is assumed to be one of the possible mechanisms of endothelial dysfunction and overt atherosclerosis [22].

Also, the disability status of the disease is associated with low level of physical activity which may lead to a higher susceptibility to subclinical atherosclerosis and cardiovascular disease [23]. This may explain the reported positive correlation in our study between arterial stiffness and both disease duration and disability.

In contrast to our results, Mincu et al. assessed arterial function in MS patients by measuring carotid–femoral pulse wave velocity and augmentation index using Complior System, in addition to carotid IMT. They found that the measured parameters of arterial stiffness were similar between both the MS group and the control group. Meanwhile, similar to our findings, carotid IMT was not significantly different between the two groups [24]. On the other hand, Garett Griffith et al. reported a significant increase in carotid IMT in older MS patients, compared to the older control group, but they did not find a significant difference between the young patients and their matched controls [25].

In our study, we measured the serum lipids in MS patients and control subjects. We found significantly higher serum levels of T-cholesterol (*P*-value < 0.001), triglycerides (*P*-value = 0.25) in MS patients, compared to controls. Meanwhile, serum levels of LDL-C and VLDL-C were insignificantly higher in MS patients, compared to controls, but serum levels of HDL-C were significantly lower in MS patients, compared to controls (*P*-value = 0.007). We did not find a significant correlation between T-cholesterol, triglycerides, or LDL-C and disease duration or disability.

Similar to our findings, Soliman et al. reported significantly higher serum levels of both LDL-C and triglycerides, and significantly lower serum levels of HDL-C in MS patients compared to controls (*P*-value = 0.001, 0.02 and 0.01, respectively), but they did not find a significant correlation between lipid profile in MS patients and disease duration or disability. They also reported an increased prevalence of insulin resistance (IR) among MS patients, compared to controls, suggesting that IR might have contributed to the reported impaired lipid metabolism [26].

Moreover, Sayonara Rangel et al. reported significantly higher serum levels of LDL-C and triglycerides, and significantly lower levels of HDL-C in MS patients, compared to controls **(***P*-value = 0.015**,** 0.025 and 0.025, respectively), but there was no significant correlation between serum lipids and disease disability. The authors also assessed the serum levels of inflammatory cytokines: IL-6 and IL-17, which were found to be correlated with the levels of serum lipids, suggesting the involvement of the inflammatory status of MS in the pathogenesis of impaired lipid metabolism [27].

Many mechanisms were suggested to explain this relationship between multiple sclerosis and dyslipidemia. The association between inflammation and alterations in lipid metabolism is considered one of the well-established mechanisms [28]. Inflammation-induced modifications of HDL-C are shown to affect its function with a reduced capacity of reverse cholesterol transportation [29]. Also, many studies reported an association between the decreased insulin sensitivity in MS patients and lipoprotein abnormalities [30]. Another hypothesis is that elevated serum lipids in MS may occur as a secondary by-product of myelin destruction in the central nervous system [31].

Disconcordant with our work, Selçuk Comoğlu et al. found that levels of total cholesterol were insignificantly higher in MS patients compared to controls. HDL-C and LDL-C levels were not statistically different between the MS group and the controls. However, similar to our results, the levels of triglycerides were significantly higher in the MS group, compared to healthy subjects [32]. Likewise, Navarro et al. found that the mean levels of plasma total cholesterol, HDL-C, and triglycerides were not significantly different between MS patients and controls [33].

Also, in contrast to our findings, some studies demonstrated an association between dyslipidemia and disability in MS patients. Tettey et al. found that nearly all lipid-related variables were positively correlated with baseline EDSS and the subsequent change in EDSS [34].

Also, Bianca Weinstock-Guttman et al. found that EDSS worsening was associated with higher baseline LDL-C, T-cholesterol, and triglycerides, while higher HDL-C levels were associated with lower contrast-enhancing MRI lesion volume. They suggested that dyslipidemia may increase disease progression by activation of the inflammatory processes at the vascular endothelium [35]. Such an association between dyslipidemia and disease progression may suggest a potential clinical benefit of lipid-lowering agents in MS.

Our study has some limitations; firstly, we only studied the vascular function in a single variant of MS, RRMS. Secondly, we did not perform clinical or neurophysiological assessment of autonomic function in MS patients, so we could not study the relationship between cardiovascular dysautonomia and arterial stiffness. Thirdly, we did not assess the levels of inflammatory mediators in MS patients and consequently, we could not establish an actual role of inflammation in vascular dysfunction in MS. The small sample size is considered also one of the limitations in our study.

## Conclusions

MS patients had significantly impaired vascular function (assessed by the brachial cuff-based method via Mobil-O-Graph device) in comparison with control subjects. Vascular dysfunction was significantly correlated with disease duration and disability. Moreover, patients with MS had significantly higher levels of T-cholesterol and triglycerides, and significantly lower levels of HDL-cholesterol, in comparison with controls. There was no statistically significant correlation between serum lipids and either disease duration or disability. No statistically significant difference was found between patients who received DMDs and those who did not receive DMDs, regarding the vascular function or serum lipids.

## Data Availability

The datasets used and/ or analyzed during the current study are available from the corresponding author on reasonable request with permission of Faculty of Medicine, Beni-Suef University, Egypt.

## Abbreviations

AIX: Augmentation index
BMI: Body mass index
BSA: Body surface area
CV: Cardiovascular
CIMT: Carotid intima-media thickness
CCA: Common carotid artery
DBP: Diastolic blood pressure
DMDs: Disease-modifying drugs
ESC: European society of cardiology
EDSS: Expanded Disability Status Scale
FDA: Food and Drug Administration
HR: Heart arte
HDL-C: High-density lipoprotein cholesterol
IR: Insulin resistance
IFN: Interferon
IL: Interleukin
ICA: Internal carotid artery
LDL-C: Low-density lipoprotein cholesterol
MRI: Magnetic resonance imaging
MS: Multiple sclerosis
PWV: Pulse wave velocity
RRMS: Relapsing–remitting multiple sclerosis
SD: Standard deviation
SBP: Systolic blood pressure
T-cholesterol: Total cholesterol
TC: Total cholesterol
TGs: Triglycerides
TNF: Tumor necrosis factor
VLDL-C: Very-low-density lipoprotein cholesterol
